# Bone healing study of alendronate combined with enoxaparin sodium bone cement in rabbits with bone defects

**DOI:** 10.1186/s13018-022-03330-y

**Published:** 2022-09-29

**Authors:** Zhihang Xiao, Dehao Fu, Li Zhang, Weiye Fan, Xiaoyu Shen, Xiangbei Qi

**Affiliations:** 1 Department of Orthopaedic Surgery, The Third Hospital of Hebei Medical University, Shijiazhuang, 050035 People’s Republic of China; 2 Department of Orthopedics, Shanghai General Hospital, Shanghai Jiao Tong University School of Medicine, Shanghai, People’s Republic of China

**Keywords:** Enoxaparin sodium bone cement, Alendronate, Bone defect, Bone cement

## Abstract

**Background:**

To observe the effect of enoxaparin sodium-polymethyl methacrylate (ES-PMMA) bone cement supplemented with alendronate (AN) on bone repair of bone defects in New Zealand rabbits.

**Methods:**

Twenty-seven New Zealand rabbits were randomly divided into ES/AN, ES-PMMA and PMMA groups, with a total of 27 New Zealand rabbits. The drugs loaded in 40 g bone cement powder were as follows: ES/AN group 8000 AxaIU enoxaparin (ES) and 200 mg alendronate (AN), ES-PMMA group 8000 AxaIU enoxaparin (ES), PMMA group without drugs. A bone defect model with a length of 10 mm and a diameter of 5 mm was made from the left tibia of rabbits, and the prepared bone cement was placed in the tibia defect. At 4 weeks, 8 weeks and 12 weeks after the operation, 3 rabbits in each group were sacrificed, and left tibia samples were collected for histological scoring, HE staining and Masson staining. Bone mineral density and new bone volume were measured by imaging, and the related data were processed by one-way ANOVA and least significance difference (LSD) post hoc test.

**Results:**

(1) Bone mineral density (BMD, mg/mm3) around the bone defect: at the 4th week, BMD in the ES/AN group was higher than that in the PMMA group; at the 8th week, the BMD in the ES/AN group was significantly higher than that in the other two groups; and at the 12th week, the BMD in the ES/AN group was significantly higher than that in the other two groups. (2) New bone volume (BV, mm3): at the 4th week, BV in the ES/AN group was significantly higher than that in the other two groups, BV in the ES/AN group was significantly higher than that in the other two groups at the 8th and 12th weeks, and BV in the ES-PMMA group was higher than that in the PMMA group. (3) Histological score: at the 4th and 8th weeks, the histological score of the ES/AN group was higher than that of the PMMA group, and at the 12th week, the histological score of the ES/AN group was higher than that of the other two groups. (4) Cortical bone thickness (μm): at the 4th, 8th and 12th weeks, the cortical bone thickness in the ES/AN group was higher than that in the other two groups, and the cortical bone thickness in the ES-PMMA group was higher than that in the PMMA group. (5) The percentage of mature area of new bone in the ES/AN group was higher than that in the other two groups at the 4th week, and at the 8th and 12th weeks, the percentage of mature area of new bone in the ES/AN group and ES-PMMA group was significantly higher than that in the PMMA group.

**Conclusion:**

(1) Enoxaparin sodium bone cement supplemented with alendronate was superior to enoxaparin sodium bone cement and PMMA bone cement in promoting bone repair of tibial bone defects in New Zealand rabbits. (2) Enoxaparin sodium bone cement is superior to PMMA bone cement in promoting bone repair, showing a certain osteogenic potential.

## Background

Polymethyl methacrylate (PMMA) bone cement is a biomaterial originally used as an adhesive in hip and knee arthroplasty. Buchholz took the lead in mixing antibiotics into bone cement, and the experiment proved that antibiotics could be released from bone cement to achieve antimicrobial concentration [1]. The release of drug-loaded bone cement conforms to the diffusion principle, and water-soluble drugs are released in a "random and irregular" manner [2]. Inspired by the application of antibiotic bone cement, different drugs are added to bone cement, which are loaded into the body through bone cement and exert local effects. In China, the prevalence of osteoporosis in adults over 40 years old is 5.0% in males and 20.6% in females [3]. Alendronate (AN) is the most common oral anti-osteoporosis drug. Its disadvantage is poor gastrointestinal absorption, which is generally only 1%[4], and its excretion rate in the kidney reaches 38–73% [5]. Song et al. [6] proved that AN could be loaded in bone cement and released at the site of bone cement placement to inhibit the activity of osteoclasts. In the range of less than 500 mg, the inhibition of osteoblast apoptosis increased with increasing dose. Second, alendronate can be continuously released in the simulated body fluid environment. The literature shows that the cumulative release rate of alendronate can still be observed within 90 days after the placement of bone cement carriers containing alendronate [7]; that is, alendronate can be continuously released with the extension of time in a certain period of time. For patients with lower extremity and spinal fractures, anticoagulant therapy with low molecular weight heparin is indispensable, among which enoxaparin sodium (ES) is the most widely used [8]. Sun et al. [9, 10] studied the release characteristics of PMMA bone cement loaded with ES. In their study, the amount of enoxaparin sodium added was divided into 4000, 8000, 12,000, 16,000, 20,000 and 24,000 AxaIU. When the amount of ES added was 8000 AxaIU, ES could not only be released from bone cement normally but also the drug concentration could reach the therapeutic dose within 24 h. After 24 h, the drug concentration was maintained between the prophylactic dose and therapeutic dose, so the addition of 8000 AxaIU enoxaparin sodium could exert a local anticoagulant effect. However, when the dosage of enoxaparin sodium exceeds 8000 AxaIU, the concentration of the drug released within 24 h will exceed the maximum dose of anticoagulant therapy, which may cause bleeding risk. Therefore, 8000AxaIU is considered a safe and appropriate dosage to add to bone cement. Meanwhile, low molecular weight heparin has been shown to have binding affinity with many growth factors, such as vascular endothelial growth factor (VEGF) [11]. Biomaterials containing heparin have been shown to have advantages in controlling the release of these growth factors [12]. The concentration of 30 mg/g [13] is conducive to the proliferation of human bone marine-derived stromal cells (hBMSCs), and heparin mainly stabilizes the prevascular structure in bone defects so that it is not easy to degrade in the microenvironment. Kim et al. [14] inhibited the expression of the proinflammatory cytokines interleukin-6 (IL-6) and tumor necrosis factor (TNF-ɑ) by loading heparin on bone substitutes in vitro. Thourani et al. [15] demonstrated that low molecular weight heparin inhibited TNF-stimulated activation of human endothelial cell lines. Further studies have shown that low molecular weight heparin downregulates proinflammatory cytokines in lipopolysaccharide (LPS)-stimulated human monocytes [16]. On the other hand, in the study of Cao [17] et al., routine subcutaneous injection of enoxaparin sodium increased the expression of BMP-2 and VEGF, resulting in better results and angiogenic activity. At the same time, low molecular weight heparin can be loaded into bone implants as an osteogenic delivery system to promote bone repair [18]. Enoxaparin sodium, as a kind of low molecular weight heparin, has the theoretical condition of promoting new bone formation in the process of bone repair. In conclusion, the release of enoxaparin and alendronate from bone cement loaded separately has been verified. We considered whether the direct and indirect positive effects of alendronate and enoxaparin on bone repair could be used to accelerate the speed of bone repair to achieve the purpose of treatment. New Zealand rabbits were sampled at 4, 8, and 12 weeks. The reasons for choosing these three time points were as follows: First, fracture healing was divided into three stages: hematoma organizing stage, callus formation stage, and callus remodeling stage. Among them, the hematoma organizing period is generally 2–3 weeks, the callus formation period is generally 4–6 weeks, and the callus remodeling period is at least 6 months. The time of animal euthanasia is roughly in line with the transformation of the three stages of fracture healing, which can accurately reflect the characteristics of bone healing in different periods. Second and most critically, the release of drugs loaded by bone cement has been confirmed to be sustained in previous literature, and the longest sustained release time in the literature can reach 90 days [7]. However, it is worth noting that during the release process, in the early stage of drug release (approximately 4 weeks), the release of the drug accounted for 50% of the total release. Weeks 4, 8, and 12 are also the cut-off points for drug release, which can reasonably evaluate the effect of drug release in the same time period. At the same time, these three time points ensure that the drug will not be released prematurely or completely or that the drug release rate is still accelerating, leading to uncontrollable results. We designed the defect size as a critical size bone defect of 10 mm in length and 5 mm in width. Critical size was defined as the smallest bone defect that could not repair itself in a lifetime. Nonunion is related to the site and degree of injury, so defects larger than the site of injury with cortical circumference greater than 50% are usually defined as critical defects [19]. We chose the tibia as a model because it has less blood supply and surrounding soft tissue, which is more likely to meet the condition of critical defects. Experimental studies on critical size bone defects have been carried out under the conditions of 8 mm and 10 mm [20], so we defined the size of tibial defects as 10 mm long and 5 mm wide. To observe the effect of the new enoxaparin/alendronate cement and enoxaparin sodium cement on bone repair in New Zealand rabbit tibial bone defects. To investigate whether enoxaparin/alendronate cement and enoxaparin sodium cement have the potential to promote bone repair in bone defects compared with PMMA bone cement.

## Materials and methods

### Experimental materials

Polymethyl methacrylate bone cement (Heraeus, Germany, 40 g/suit); enoxaparin sodium freeze-dried powder (Chengdu Baiyu Pharmaceutical Co., Ltd., 4,000 AxaIU/ branch, national medicine brand: H20150010); 3D printing standard size bone cement mold (Canghai 3D Printing Co., Ltd.), which meets the requirements of ISO: 2002 “Surgical Implant-Acrylic Resin Bone cement” Masson dyeing solution (Beijing Solebao Technology Co., Ltd.); hematoxylin dye, eosin dye (Zhuhai Beisuo Biotechnology Co., Ltd.); differentiation solution (Shanghai Biyuntian Biotechnology Co., Ltd.), and xylene, anhydrous ethanol and neutral gum (Tianjin Yongda Chemical Reagent Co., Ltd.). A 4% paraformaldehyde solution, PBS equilibrium salt solution, EDTA decalcification solution, 25% urethane solution (Hangzhou Aikai Medical Supplies Co., Ltd.); alendronate (stone pharmaceutical group Ouyi Pharmaceutical Co., Ltd., 10 mg/tablets); microcomputer tomography (model Quantum, manufacturer PerkinElmer, origin United States); paraffin slicer (model RM2135, Leica Germany). Optical microscope (model BX43, Japanese OLYMPUS company) and imaging system (model UC90, Japanese OLYMPUS company).

### Experimental animals

Male New Zealand rabbits with 6-month-old body weights of 2.5–3.2 kg were purchased from the Animal Experimental Center of the Third Hospital of Hebei Medical University and bred in Tonghui County, Wangdu County. The animal certificate number is 211105.

### Experimental grouping

The experiment was divided into ES/AN, ES-PMMA and PMMA groups, with 9 rabbits in each group. The 40 g bone cement powder loaded with drugs were as follows: ES/AN group 8000 AxaIU enoxaparin (ES) and 200 mg alendronate (AN), ES-PMMA group 8000 AxaIU enoxaparin (ES), PMMA group did not add drugs.

### Sample preparation

All the experimental steps of sample preparation were carried out in a standard clean operating room environment with a room temperature of approximately 23 °C and a humidity of approximately 40–60%. Alendronate sodium tablets and enoxaparin sodium freeze-dried powder were ground into fine powder in an aseptic open container before the experiment. Preparation of ES/AN bone cement: add 200 mg alendronate and 8000 AxaIU unit enoxaparin sodium into 40 g bone cement powder and mix it well [21, 22], add liquid monomer to stir quickly, put into the prepared cylinder photosensitive resin mold of approximately 10 mm and diameter 5 mm at dough stage [23], wait for the bone cement to enter the curing period, take out the bone cement column, and check the size of bone cement with Vernier caliper. The bone cement with bubbles and cracks on the surface and inside was excluded by visual and X-ray. The preparation method of bone cement in the ES-PMMA group and PMMA group was the same as before.

### Surgical modeling

**Fig. 1:**
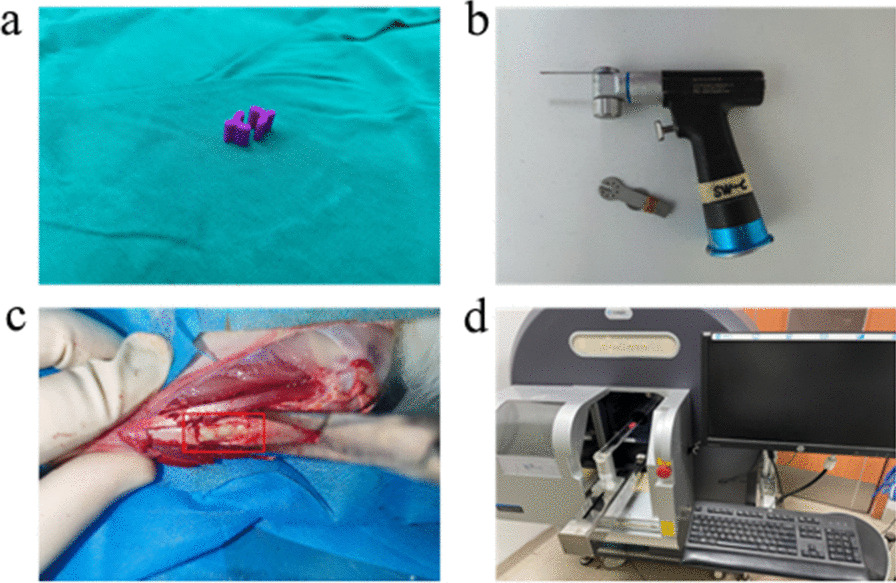
Relevant equipment required during the experiment. **a** Bone cement module with a length of 10 mm and a diameter of 5 mm. **b** Bone saw used to create the same size bone defect. **c** Bone cement of standard size is placed on the bone defect. The bone cement module is placed in the red box. **d** Microcomputed tomography (micro-CT) scan of the tibia specimen

All animal use procedures for surgical modeling were based on the guidelines for Laboratory Animal Welfare of Hebei Medical University and approved by the Experimental Animal Ethics Committee of the Third Hospital of Hebei Medical University. All animals were raised under standard conditions and allowed to adapt to the environment for one week. urethane (25%) was anesthetized through the ear vein. Before the operation, the rabbit hair of the test epidermis of the left tibia was shaved off with a shaver, the operation area was disinfected with iodine alcohol, and a longitudinal 50 mm incision was made along the medial side of the tibia. After the skin and muscle tissue were separated, the periosteum was removed, and 5 mm on the medial side of the left tibial tubercle was selected. A unilateral cortical bone defect 10 mm long and 5 mm wide was made with a bone saw (Fig. 1). The defect was continuously washed with 0.9% sodium chloride aqueous solution during the operation, the bone cement module was fixed on the defect with absorbable suture, the wound was sutured layer by layer with nonabsorbable thread, and the dressing was bandaged after injury. No rabbits died during the operation, all rabbits were allowed to move freely after the operation, and 80,000 units of penicillin were injected intramuscularly after the operation to prevent infection. The animals were fed normally after the operation, and the animals were killed under anesthesia at 4, 8 and 12 weeks. The left tibia was removed under aseptic conditions and preserved in 4% paraformaldehyde solution.

### Imaging evaluation

**Fig. 2:**
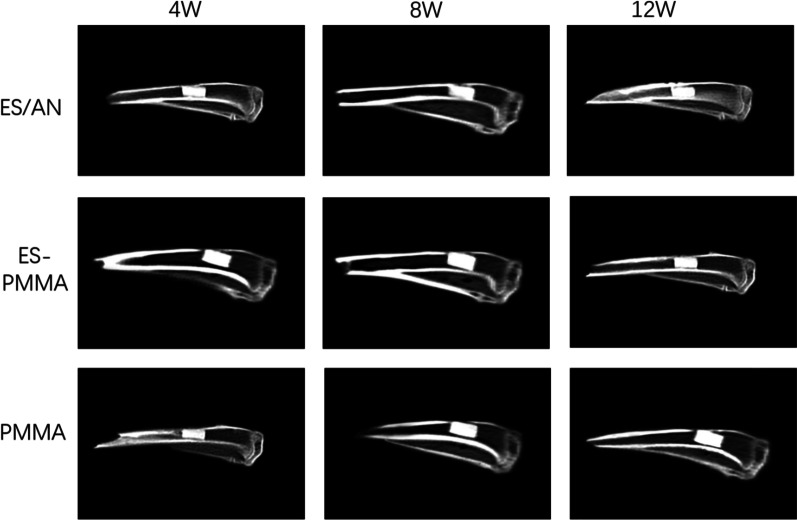
Computed tomography scan of tibial bone defect at 3 time points in each group. At 4 weeks, 8 weeks and 12 weeks after the end of the experiment, the tibial bone defect was scanned by computed tomography

The tibia specimen was stored in 4% paraformaldehyde solution, and the range of 10 mm and distal and proximal 3 mm at the bone defect was set. The region with a total length of 16 mm and a width of 5 mm was the region of interest (ROI). Micro-CT scanning was performed under the following conditions: voltage 60 kV, current 500 μA. Bone histomorphometry and three-dimensional reconstruction were performed by a high-resolution computed tomography system in all scanning planes, and bone mineral density (BMD, mg/mm^3^) was measured. The bone mineral density (BMD) was calculated as the average value in the ROI. The BMD of each sample at the proximal and distal ends of the defect was measured three times at each time point, and the average value of each group was calculated. The most obvious part of the defect was photographed at the appropriate cross-section of the ROI (Fig. 2). The ROI area of the specimen was analyzed by a data analyzer (CaliperAnalyze), and the new bone volume (BV, mm^3^) was measured and recorded.

### Histological staining

Tibial specimens were soaked in 4% paraformaldehyde solution for 24 h. The specimens were rinsed 3 times with PBS and then decalcified in 10% EDTA decalcification solution (pH 7.2) for 3 weeks, and the decalcification solution was changed every 24 h. After dehydration in gradient ethanol solution and treatment with an embedding machine, the tissue sections were prepared to a final thickness of 4 microns by a paraffin microtome and then stained with hematoxylin and eosin (HE) and Masson staining. The sections were examined with a light microscope at magnifications of 50×, 100× and 200×. Histological scores were evaluated by 7 experienced pathologists, and the scoring criteria were uniformly assigned to 7 physicians. After all 7 pathologists understood and had no objection, each physician individually scored the histological sections (Table 1). After obtaining the scores, each section was averaged and recorded. The contact interface between the bone cement and surrounding bone tissue was observed in a 100 × visual field. Seven different areas were randomly selected on the bone-cement contact surface for each section, and the cortical bone thickness (µm) and the percentage of new and mature bone area (%) were measured by ImageJ (version 1.52K) software. The quantitative analysis of cortical bone thickness was performed by determining the extent of new bone, converting the color frame to a gray 8-bit depth map, calculating the area S by the Analyze module function, and measuring the Length L by Length. The calculation formula was cortical bone thickness = S/L, which was measured by three researchers skilled in using the software, and the average value was taken and recorded. Masson staining was applied to the collagen staining. At different time points, the collagen in the bone matrix was red and blue, and the mature bone tissue (lamellar bone) was stained red, while the immature bone tissue (woven bone) was stained blue. The percentage of mature area of new bone was determined by staining and analyzing particle function data analysis with the Analyze module in ImageJ software.

**Table 1:** Histopathological score [24]

Healing of defect area	Score
Without any reaction	0
Fibrosis with inflammation	1
Fibrous tissue	2
Mainly fibrous tissue, poor formation of braided bone	3
Mainly braided bone formation, poor fibrous tissue	4
Focal fibrosis and/or braided bone combined with lamellar bone formation	5
Only flaky bone texture (fully healed)	6

### Statistical analysis

Continuous variables were expressed as mean ± standard deviation $\left( {{\overline{\text{x}}} \pm {\text{s}}} \right)$. Kolmogorov–Smirnov test was used for the normality test. One-way ANOVA (ANOVA) was used to analyze the differences between groups, and least significance difference (LSD) test was performed for post hoc comparisons. A two-tailed *p* value < 0.05 was considered to be statistically significant for all tests. All statistical analyses were performed using SPSS 26.0 (IBM Company, Armonk, NY, USA).

## Result

### General observation

In the fourth week, when the original wound was opened and the bone defect was exposed by the separated tissue, the induced membrane was visible on the surface of the bone cement, and the distal and proximal ends of the induced membrane exceeded the size of the bone defect, extended to the distal and proximal bone surface and contacted the bone cortex.

### Imaging examinations

**Fig. 3:**
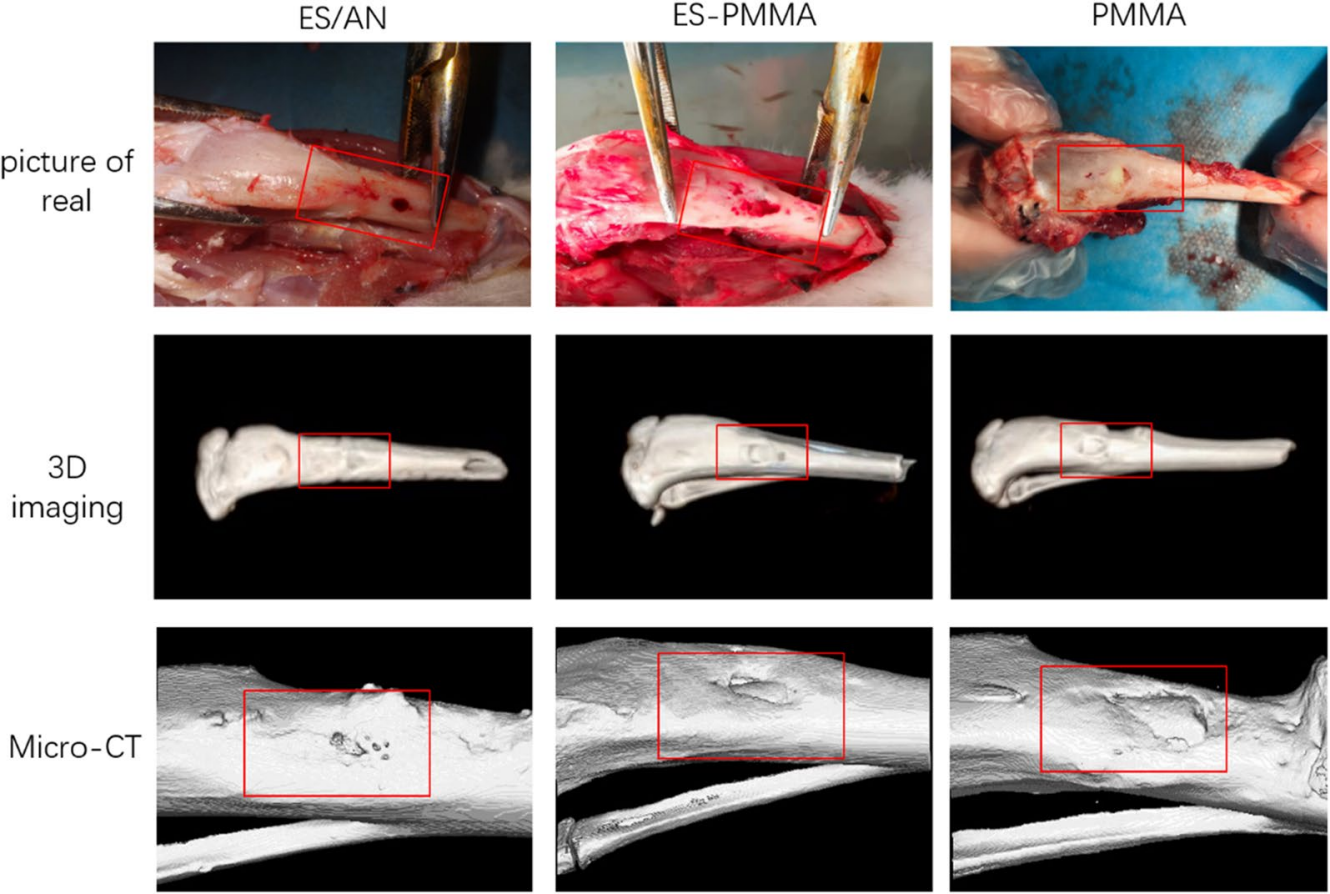
At the 12th week, animal samples, microscopic CT scans and 3D imaging were compared among the three groups. The red box shows the area where the bone defect was created

**Fig. 4:**
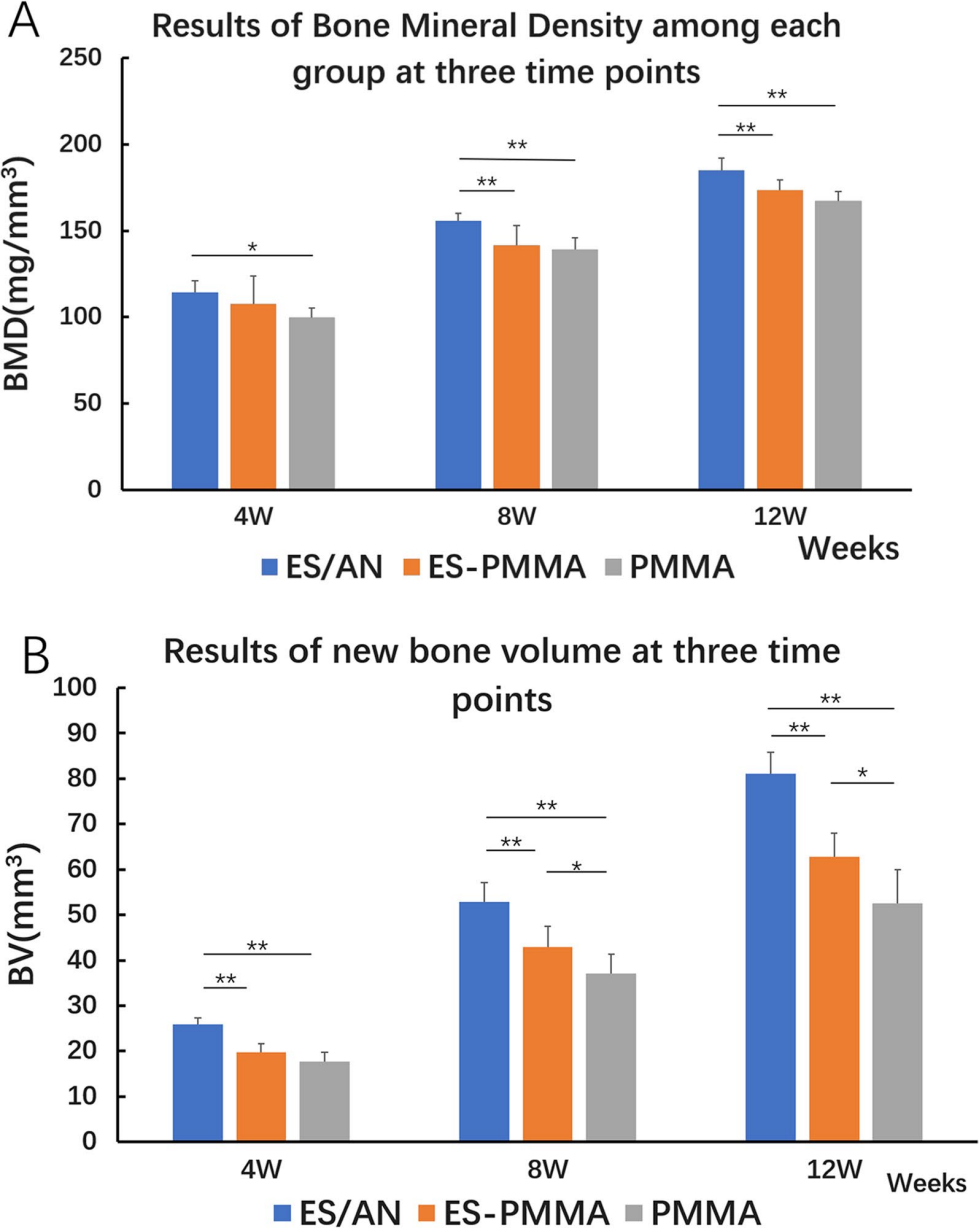
Imaging data results at three time points in each group. Imaging analysis of bone formation among the three groups: **A** Bone mineral density: At each time point, bone mineral density was measured three times near and far from the defect for each sample to calculate the mean value in the selected area. **B** New bone volume: the volume of new bone in the selected area. The selected area (ROI) was 16 mm long and 5 mm wide, including 10 mm at the bone defect and 3 mm near and far. Statistical differences between the three groups are shown (one-way ANOVA followed by LSD test for post hoc comparisons,*p < 0.05; **p < 0.01)

The degree of bone repair of rabbits with bone defects at 4, 8 and 12 weeks after implantation of bone cement was observed by microcomputer tomography (Fig. 3). Compared with the pictures at each time point, the gray area of ES/AN bone cement was the highest, and the bone calcification density was the highest in the bone defect, followed by the ES-PMMA group and the PMMA group. At the 4th week, the bone mineral density around the bone defect in the ES/AN group was higher than that in the PMMA group, that in the ES/AN group was higher than that in the ES-PMMA group and PMMA group, and that in the ES/AN group was higher than that in the ES-PMMA group and PMMA group at the 12th week. At the 4th week, the new bone volume (BV, mm3) in the ES/AN group was higher than that in the ES-PMMA group and the PMMA group, respectively, and at the 8th and 12th weeks, it was the highest in the ES/AN group, the second in the ES-PMMA group, the lowest in the PMMA group, and significantly higher in the ES/AN group than that in the other two groups, while that in the ES-PMMA group was higher than that in the PMMA group (Fig. 4).

### Histological expression

**Fig. 5:**
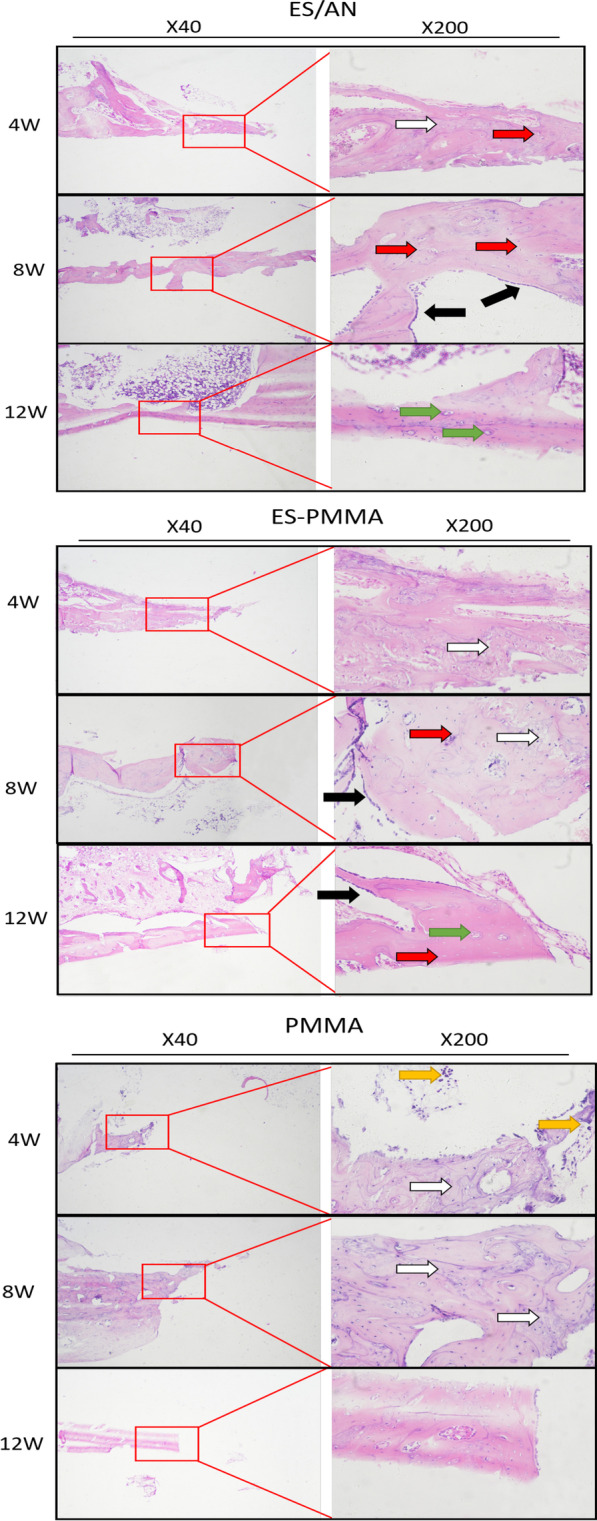
HE staining in each group at three time points. After 4 weeks, 8 weeks, and 12 weeks of the experiment, the HE-stained images of the defect areas were observed under a light microscope at ×40 and ×200 magnification. Black arrows indicate areas of inflammation and green arrows indicate neovascularization. Black arrows—Indicates osteoblast, yellow arrows—Indicates Inflammatory cells, green arrows—Indicates neovascularization, red arrows—Indicates Bone cells, white arrows —Indicates Immature bone tissue

**Fig. 6:**
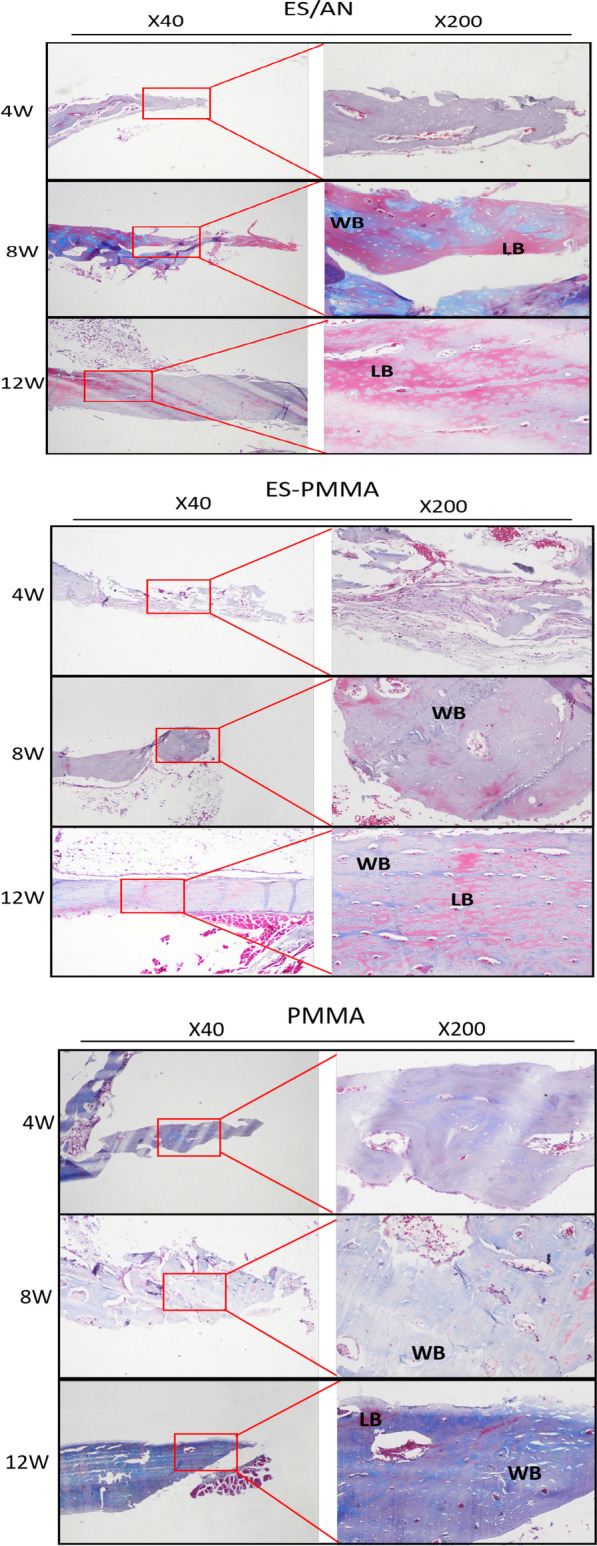
Masson staining was performed at three time points in each group. After 4, 8 and 12 weeks of experiment, Masson staining images of the defect areas were observed under light microscopy at ×40 and ×200 magnification. LB: Lamellar Bone, which is newly formed mature bone tissue. WB: Woven Bone, an immature bone tissue dominated by collagen fibers

**Fig. 7:**
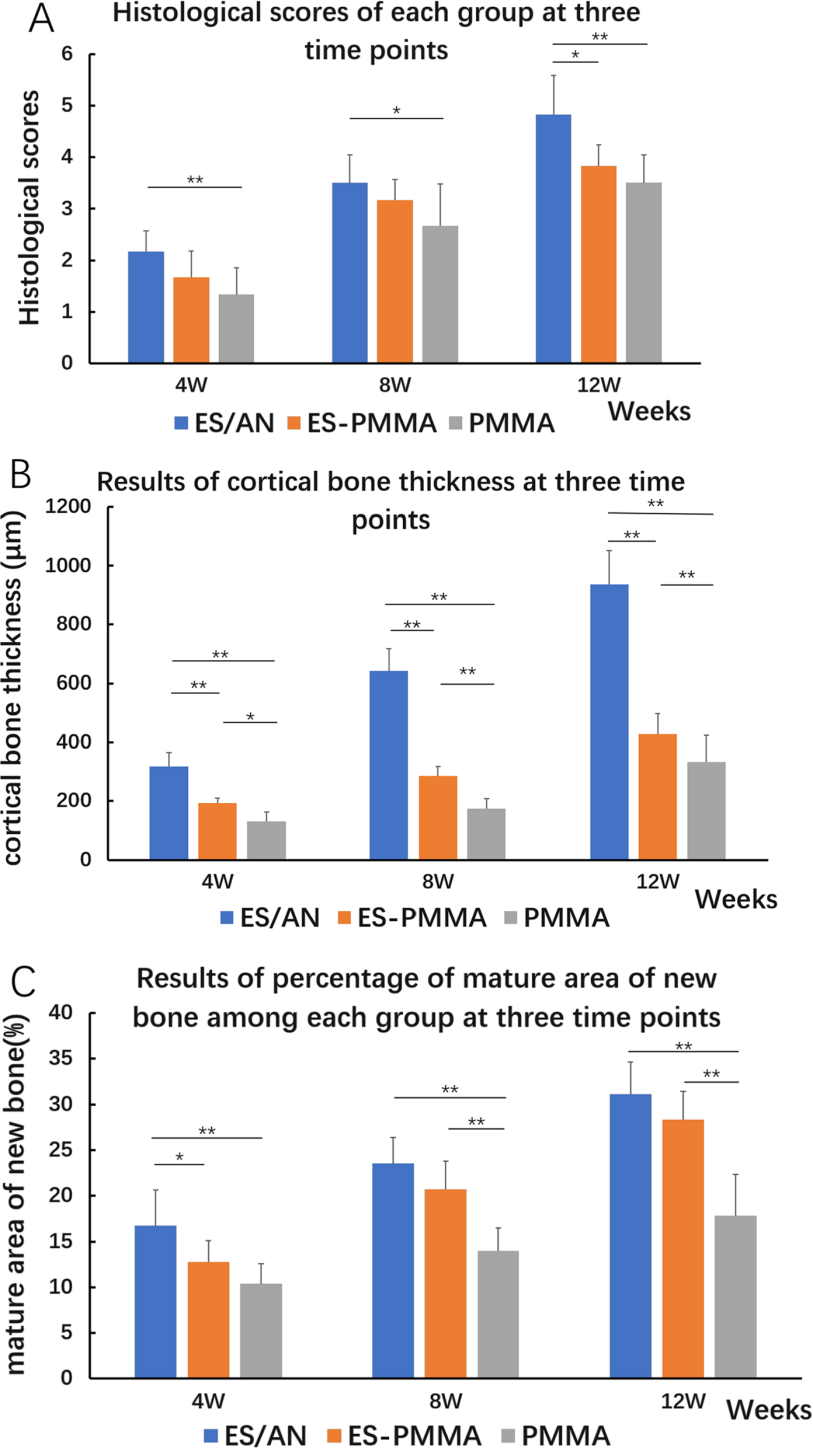
Histological data were obtained for each group at three time points. Histological analysis of bone formation among the three groups: **A** Histological score: The sections were observed under the light microscope in accordance with Table 1 for parallel histological score. **B** Cortical bone thickness: the mean value of cortical bone thickness in 7 fields randomly selected at the bone cement interface of the defect was measured. **C** Percentage of mature area of new bone: The percentage of lamellar bone in the image was measured by ImageJ software at ×100 magnification at the interface between bone and bone cement at the defect. Statistical differences between the three groups are shown (one-way ANOVA followed by LSD test for post hoc comparisons,*p < 0.05; **p < 0.01)

In HE (Fig. 5) and Masson (Fig. 6) staining, the number of inflammatory cells at the edge of the bone defect in the ES/AN group was less than that in the ES-PMMA group and PMMA group at the 4th week, and the number of new tissues in the PMMA group was greater than that in the PMMA group. At 8 weeks, the distal and proximal bone cortex began to connect in the ES/AN group, and obvious newly formed bone tissue was seen in the ES-PMMA group, but not as much as in the ES/AN group, but not in the PMMA group. At 12 weeks, there were continuous bone tissue covering defects and obvious bone mineralization on the interface between bone and cement in the ES/AN group. This result is basically consistent with the results observed by imaging. The histological score of the ES/AN group was better than that of the PMMA group at the 4th and 8th weeks. At the 12th week, the ES/AN group was superior to the ES-PMMA group and PMMA group. The cortical bone thickness (μm) was the highest in the ES/AN group, the second in the ES-PMMA group and the lowest in the PMMA group. At the 4th week, the percentage of mature area of new bone in the ES/AN group was higher than that in the ES-PMMA group and PMMA group. At the 8th and 12th weeks, the data of the ES/AN group and ES-PMMA group were better than those of the PMMA group, but there was no significant difference between the ES/AN group and ES-PMMA group (Fig. 7).

## Discussion

In the imaging results, the BMD in the ES/AN group was significantly higher than that in the PMMA group at 4, 8 and 12 weeks, but there was no significant difference in local BMD between the ES-PMMA group and PMMA group at the three time points. The increase in bone mineral density is related to the two local effects of alendronate. On the one hand, AN is released from bone cement, and alendronate around the bone defect is retained in the place with high bone turnover through its affinity with minerals in the bone extracellular matrix, inhibiting the activity of osteoclasts, that is, inhibiting bone resorption, thus making bone formation greater than bone resorption and increasing bone mineral density [25]. On the other hand, the concentration of alendronate did not inhibit the viability and proliferation of osteoblasts. In the study of Rumian et al. [26], alendronate had no significant effect on the MG63 activity of osteoblast-like cells when the concentration of alendronate was 5 mg/ml and could reduce the differentiation of monocytes from peripheral blood lymphocytes to osteoclasts. The results of imaging new bone volume showed that there was no significant difference between the ES-PMMA group and PMMA group at the 4th week, but there was a significant difference among the other groups at other time points. The new bone volume (BV) results showed that the ES/AN group was better than the ES-PMMA group and PMMA group at each time point. At the 8th and 12th weeks, the volume of new bone formation in the ES-PMMA group was larger than that in the PMMA group, indicating the promoting effect of enoxaparin sodium on osteoblasts with local application. This is consistent with the in vitro study of Rodriges et al. [27], which confirmed that low molecular weight heparin stimulated the osteogenic differentiation of human dental pulp cells, increased the activity of alkaline phosphatase, increased the expression of bone morphogenetic protein (BMP-2) and osteocalcin, and promoted the formation of mineralized nodules in the extracellular matrix, reflecting the promoting effect of local application of low molecular weight heparin on new bone formation.

In histological staining, the thickness of cortical bone is the average value measured many times after histological staining. There were significant differences in cortical bone thickness among the three groups: the ES/AN group was the best, the ES-PMMA group was the second, and the PMMA group was the lowest. The percentage of mature area of new bone in the ES/AN group was higher than that in the PMMA group at the three time points. There was a significant difference between the ES-PMMA group and PMMA group at the 8th and 12th weeks, but there was no significant difference between the ES/AN group and ES-PMMA group at the 8th and 12th weeks. The transformation of braided bone to lamellar bone in the early ES/AN group was faster than that in the ES-PMMA group. Over time (8 weeks and 12 weeks), the trend of bone maturation in the ES-PMMA group was faster than that in the PMMA group. In HE staining and Masson staining, the number of osteoblasts around the new bone in the ES/AN group was greater than that in the ES-PMMA group and PMMA group, indicating that alendronate had no significant negative effect on osteoblasts at the concentration of alendronate 5 mg/ml, but it should be noted that the number of osteoblasts observed by staining was only the image of bone cement in contact with surrounding tissue randomly selected by slices and was visually counted by light microscopy. There will be a slight deviation in reality.

We scored the bone samples histologically without any response to lamellar bone formation, which reflected their healing status. These stages involve inflammation, fibrosis formation, braided bone development, and finally the integration of lamellar bone development. Histological scores reflect the biological response of bone cement at bone defects and the quality of new bone and bone growth. The score of the ES/AN group was better than that of the PMMA group at the three time points and that of the ES/AN group was better than that of the ES-PMMA group at the 12th week, while there was no significant difference between the ES-PMMA group and PMMA group at each time point. At the 4th week, more inflammatory cells were observed in the PMMA group than in the ES/AN group and ES-PMMA group, while a lower inflammatory reaction was observed in the ES/AN group and ES-PMMA group. It can be seen that the fibrous capsule formed between PMMA bone cement and bone was the influencing factor hindering new bone attachment and bone repair [28]. In the Masson staining images, the bone matrix layers of the ES/AN group and ES-PMMA group were neatly arranged, indicating that the ES/AN group can not only promote new bone formation but also promote bone maturation at the site of the bone defect. There was no significant difference in the mature area of new bone between the ES-PMMA group and the ES/AN group at the 8th and 12th weeks, but there was less new bone and lower bone maturity in the PMMA group. Osteocytes and blood vessels were observed in the ES/AN group, and blood vessels were also observed in the new bone tissue in the ES-PMMA group, while in the PMMA group, the new tissue was mainly immature bone tissue with few blood vessels, indicating that enoxaparin sodium could promote bone maturation at appropriate concentrations. In the animal experiment of Chiodelli et al. [29], low molecular weight heparin was involved in the molecular mechanism of angiogenesis at a low concentration (30 mg/g). The main mediators of angiogenesis are endothelial growth factor (VEGF) and fibroblast growth factor (FGF1, FGF2), which are regulated by enoxaparin sodium and enhance their effects, thus promoting the growth of the local capillary network from peripheral endothelial cells to defects [30].

According to the results of imaging and histology, in the repair of rabbit tibial bone defects, the promotion of new bone formation in the ES/AN group (BV) was significantly better than that in the ES-PMMA group and PMMA group, while the cortical bone thickness was randomly selected and measured in software, which indirectly reflected the general trend of new bone formation. The volume of new bone calculated by three-dimensional reconstruction has the same trend as that of histological measurement, which shows that the ES/AN group has the advantage of new bone formation compared with the ES-PMMA group and PMMA group. At the same time, the volume of new bone in the ES-PMMA group was also higher than that in the PMMA group for a long time (8 weeks and 12 weeks), indicating that the ES-PMMA group had a certain osteogenic potential compared with the PMMA group, while in Masson staining, braided bone and lamellar bone could be distinguished by staining. Blue staining represents woven bone, also known as fibrous bone, which is a kind of immature bone. The red-stained area is lamellar bone, which changes from woven bone to a mature plate in the process of bone repair.

In summary, a defect that cannot heal itself under the critical size will lead to the destruction of vascular continuity and lead to bone nonunion. Lu et al. [31] believe that to obtain ideal bone remodeling, it is necessary to ensure sufficient stability of the defect. Without the role of bone cement as cavity filling in large defects, it is difficult to achieve the mechanical strength needed for bone remodeling. Enoxaparin sodium plays an anti-inflammatory role and promotes vascularization [32]. Alendronate inhibits the activity of osteoclasts and has no negative effect on osteoblasts. Rich vascularization, inhibition of osteoclast activity and anti-inflammation after bone defects all play a role in repairing bone defects, and the results are consistent with the experimental results of Jayasree et al. [33].

## Conclusion

The results showed that the potential of ES/AN bone cement to promote bone repair was better than that of ES-PMMA and PMMA and could improve new bone formation and subsequent bone maturation in the defect area. At the same time, it was observed in our experiment that the ES-PMMA group had a certain positive effect on promoting bone repair, promoted bone maturation in the new bone area, and had no clear negative effect on osteoblasts, which created conditions for bone repair.

## Data Availability

The datasets used and analyzed during the current study are available from the corresponding author on reasonable request.

## Abbreviations

HE: Hematoxylin-eosin
CT: Computed tomography
PMMA: Polymethyl methacrylate
BMD: Bone mineral density
BV: Bone volume
ROI: Region of interest
PBS: Phosphate-buffered saline
ES: Enoxaparin sodium
AN: Alendronate
WB: Woven bone
LB: Lamellar bone
ES/AN: Enoxaparin sodium/Alendronate
ES-PMMA: Enoxaparin sodium-polymethyl methacrylate
